# CD4.CD8 ratio decrease in AIDS, explained by a molecular mimicry between African HIV-1 Nef and Notch-1. Nef as a target for vaccine and NF-Kb inhibitors (salicylate, resveratrol,curcumin, epigallocatechine-3-gallate)

**DOI:** 10.1186/1742-4690-9-S1-P24

**Published:** 2012-05-25

**Authors:** Guy MK Tran, Adrien Caprani, Laurent Gerbaud

**Affiliations:** 1 Clermont-Ferrand University, Hospital Hotel-Dieu, Public Health, Clermont-Ferrand, France; 2 Association POSITIFS, Paris cedex 18, France

## Background

The AIDS hallmark is the simultaneous fall in CD4 and rise in CD8 T lymphocytes. Interestingly, this very pathognomonic but unexplained decrease of CD4/CD8 ratio is also characteristic of a member of the EGF family, Notch-1 function (Fowlkes BJ, 2002). Calenda V (1994) found that Nef hampered drastically bone marrow progenitors cells functionality. African HIV-1 strain NDK (Spire B, 1989), which induced a fulminant AIDS killing the patient in only 15 days, decreases dramatically CD4 counts. Nef is the most abundant HIV-1 protein in infected cells (85% of mRNA). Nef is a superantigen, its action is amplified 10,000 times compared to a common antigen.

## Objective

We found previously Notch-1 in the LTR (Long Terminal Repeat) of another retrovirus [Mouse Mammary Tumor Virus (MMTV)] (Tran MKG, Eurocancer, Paris, 1999). As Nef is located also in HIV-1 LTR, we looked for Notch-1 in Nef.

## Methods

Amino Acid (AA) alignment between Epidermal Growth Factor (EGF) family members (including Notch-1) and Nef (Los Alamos HIV sequences Database, 2002).

## Results

Nef COOH-terminus of HIV-1 clade D African strains (from Congo Democratic Republic, Chad, Tanzania, Uganda, South Africa, Kenya,…), but not from other parts of the world (other non-D clades), was a perfect molecular mimetic of Notch-1: They shared a heptapeptide (7 AA) SRLAFEH. The homology between Nef (Poon AFY, 2009) and Notch (BLASTP on mouse Notch-1) chimera was 67 AA long with 4 His, 1 Cys and 1 Trp (highly significant): Nef : GWCFEVEEDTEGETNSLLHPISQHGMEDPERQVLVWRFNSRLAFEHKARLMHPEFYKNC Notch : GWLLD…FEQDSEGETNSLPHLISQHAL ANPEMQALA-HGKSRLAFEHQVRLSHLPVANNC It included the Nef LL and ED doublets precisely implicated in CD4 down-regulation and EE in β-COP recruitement(Benichou S.1994).

## Conclusions

This opens new avenues for a vaccine targeted to Nef-Notch specific to Africa, a continent devastated by AIDS and tuberculosis (in South Africa, about 60% HIV-1 infected patients had also tuberculosis).

